# Durvalumab plus Paclitaxel, with or without Capivasertib or Oleclumab, in Patients with Locally Advanced/Metastatic Triple-Negative Breast Cancer

**DOI:** 10.1158/1078-0432.CCR-25-4417

**Published:** 2026-05-26

**Authors:** Peter Schmid, Cynthia X. Ma, Yeon Hee Park, Simon Lord, Anne Armstrong, Seock-Ah Im, Shin-Cheh Chen, Ricardo Fernandes, Jacek Jassem, Piotr J. Wysocki, Yen-Shen Lu, Hwei-Chung Wang, Wei-Pang Chung, Purnima Rao-Melacini, Katrin Heider, Ross Stewart, Petra Vuković, Kyung Hae Jung

**Affiliations:** 1 Barts Cancer Institute, Queen Mary University of London, London, United Kingdom.; 2 Washington University School of Medicine, St. Louis, Missouri.; 3 Samsung Medical Center, Sungkyunkwan University School of Medicine, Seoul, Republic of Korea.; 4 Medical Sciences Division, University of Oxford, Oxford, United Kingdom.; 5 Christie Hospital NHS Foundation Trust and Faculty of Biology, Medicine and Health, University of Manchester, Manchester, United Kingdom.; 6 Seoul National University Hospital, Cancer Research Institute, Seoul National University College of Medicine, Seoul National University, Seoul, Republic of Korea.; 7 Department of General Surgery, Chang Gung Memorial Hospital, Taoyuan City, Taiwan.; 8 Schulich School of Medicine & Dentistry, Western University, London Health Sciences Centre, London, Canada.; 9 Medical University of Gdańsk, Gdańsk, Poland.; 10 Jagiellonian University – Medical College, Krakow, Poland.; 11 National Taiwan University Hospital, Taipei, Taiwan.; 12 Surgical Department, China Medical University Hospital, Taichung, Taiwan.; 13 National Cheng Kung University Hospital, College of Medicine, National Cheng Kung University, Tainan, Taiwan.; 14 Late Phase Oncology, AstraZeneca, Mississauga, Canada.; 15 Late Phase Oncology, AstraZeneca, Cambridge, United Kingdom.; 16 Translational Medicine Oncology, AstraZeneca, Cambridge, United Kingdom.; 17 Asan Medical Center, University of Ulsan, College of Medicine, Seoul, Republic of Korea.

## Abstract

**Purpose:**

Triple-negative breast cancer (TNBC) is a heterogeneous disease with high recurrence rates and poor prognosis, often requiring multiple therapies. BEGONIA was a phase Ib/II, multiarm, platform study evaluating the safety and efficacy of first-line treatment combinations with durvalumab (anti–PD-L1 antibody) for locally advanced, unresectable/metastatic TNBC (mTNBC; NCT03742102). In this study, we report results of 3 treatment arms.

**Patients and Methods:**

Eligible female participants (≥18 years with untreated, unresectable, locally advanced/mTNBC) received durvalumab plus paclitaxel or were randomized to this treatment in combination with capivasertib (pan-AKT inhibitor) or oleclumab (anti-CD73 antibody). The primary objective was safety and tolerability; secondary endpoints included objective response rate (ORR).

**Results:**

Twenty-three patients received durvalumab plus paclitaxel, 31 received capivasertib combination, and 33 received oleclumab combination. Maximum grade 3/4 adverse events occurred in 10 of 23 (43.5%), 25 of 31 (80.6%), and 8 of 33 (24.2%) patients in the durvalumab plus paclitaxel, capivasertib-combination, and oleclumab-combination arms, respectively. The confirmed ORR (95% confidence interval) was 56.5% (34.5–76.8) with durvalumab plus paclitaxel, 54.8% (36–72.7) with capivasertib combination, and 51.5% (33.5–69.2) with oleclumab combination. Responses were observed across biomarker subgroups, including PD-L1, *PIK3CA/AKT1/PTEN* alterations, and CD73, with a trend for improved activity in the PD-L1–positive subgroups.

**Conclusions:**

These findings support the clinical activity and tolerability of durvalumab plus paclitaxel in locally advanced unresectable/mTNBC, as expected for an immune checkpoint inhibitor in combination with chemotherapy. The addition of capivasertib or oleclumab to this treatment combination showed no substantial additional benefit. PD-L1 expression was associated with enhanced antitumor activity across all arms.

**Translational Relevance**

The combination of durvalumab and paclitaxel has shown clinical activity and tolerability in locally advanced, unresectable/metastatic triple-negative breast cancer, as expected for an anti–PD-L1 antibody in combination with chemotherapy. Additional PI3K/AKT/PTEN targeting or CD73 blockade may further improve outcomes. However, the addition of capivasertib or oleclumab to durvalumab and paclitaxel resulted in no substantial additional benefit. The observed safety profiles were consistent with the known profiles of each agent, with no unexpected toxicities observed. Responses were also observed across biomarker subgroups, with a trend for improved activity in the PD-L1–positive subgroups and numerical improvements in those with *PIK3CA/AKT1/PTEN* alterations and in the CD73^+^ subgroups. Although limited by the small number of patients included in these analyses, overall these results confirm that the combination of capivasertib and oleclumab with immune checkpoint inhibitor–chemotherapy in first-line treatment is unlikely to provide substantial additional benefit and align with previous studies evaluating similar therapeutic combinations.

## Introduction

Triple-negative breast cancer (TNBC) accounts for 15% to 20% of all breast cancers (1) and is defined by the lack of estrogen receptor (ER) and progesterone receptor (PR) expression and the absence of human epidermal growth factor receptor 2 (HER2) overexpression. TNBC is an aggressive disease with high recurrence rates and poor prognosis (2–4). It is characterized by high genomic instability, tumor heterogeneity, and an inflamed tumor microenvironment, highlighting the need for combination approaches that address multiple aspects of the disease biology essential for improving outcomes. Treatment with PD-1/PD-L1 inhibitors in combination with chemotherapy has demonstrated clinically meaningful improvements in progression-free survival (PFS) and overall survival (OS) versus chemotherapy alone (5, 6) in patients with PD-L1–positive, metastatic TNBC (mTNBC). Additional targeted options, including TROP2-directed antibody–drug conjugates with or without immunotherapy, have also shown efficacy in the metastatic setting (7, 8). However, there is still a need for novel treatment strategies to improve outcomes, reduce toxicity, and maintain quality of life both for patients with PD-L1–negative disease, which accounts for more than half of all TNBC cases (9, 10), and for those with PD-L1–positive disease who relapse within 10 months of receiving immune checkpoint inhibitor (ICI)/chemotherapy (5, 11, 12).

Durvalumab is a selective, high-affinity human IgG1κ monoclonal antibody (mAb; modified to remove effector function) that binds to PD-L1, overcoming PD-L1–mediated inhibition and suppression of T-cell activation (13). Several smaller studies of durvalumab in advanced TNBC suggest its therapeutic potential in this population (14–16). Notably, in an exploratory subgroup analysis of 82 patients with mTNBC eligible for first-line or second-line chemotherapy in the phase II SAFIR02-BREAST IMMUNO trial (NCT02299999), maintenance therapy with single-agent durvalumab demonstrated improved OS compared with maintenance chemotherapy [HR, 0.54; 95% confidence interval (CI), 0.30–0.97; *P* = 0.0377; ref. 14]. Durvalumab combination treatments have also demonstrated efficacy for patients with early-stage TNBC (17–19). For example, in the GeparNuevo trial, 3-year invasive disease-free survival (HR, 0.48; 95% CI, 0.24–0.97), distant disease-free survival (HR, 0.31; 95% CI, 0.13–0.74), and OS (HR, 0.24; 95% CI, 0.08–0.72) were significantly improved with neoadjuvant durvalumab monotherapy versus placebo prior to chemotherapy, despite a nonsignificant increase in the pathologic complete response (CR) rate (17).

In TNBC tumors, activation of the PI3K/AKT pathway due to an activating mutation in *PIK3CA* or *AKT1,* or *PTEN* loss, is common and has been associated with poor prognosis and outcomes (20–25). Loss of tumor *PTEN* expression has been linked to a reduction of tumor-infiltrating lymphocytes, which may in turn be responsible for a lack of ICI activity in some TNBC tumors (26). Targeting both PI3K/AKT/PTEN signaling and the PD-1/PD-L1 pathway may further improve outcomes with durvalumab. Capivasertib (AZD5363) is a potent, selective, small-molecule, ATP-competitive catalytic inhibitor of all 3 AKT isoforms (AKT1/2/3; ref. 27). The addition of capivasertib to paclitaxel 90 mg/m^2^ (days 1, 8, and 15 in 4-week cycles) resulted in significantly longer median PFS (5.9 vs. 4.2 months; HR, 0.74; 95% CI, 0.50–1.08) and OS (19.1 vs. 12.6 months; HR, 0.61; 95% CI, 0.37–0.99; 2-sided *P* = 0.04) compared with placebo plus paclitaxel as a first-line treatment for patients with mTNBC in the phase II PAKT trial (28, 29).

The confirmatory trial CAPItello-290 did not meet its primary endpoint of OS either in the overall population or in patients with *PIK3CA/AKT1/PTEN*-altered tumors (30); however, outcomes for the secondary endpoint of PFS indicated a numerical improvement in favor of the experimental arm. Of note, ipatasertib, another AKT inhibitor, has been evaluated in combination with atezolizumab and taxane as first-line treatment in the phase Ib CO40151 trial. Preliminary analysis showed a confirmed objective response rate (ORR) of 73% for the treatment combination (31), which prompted exploration of the capivasertib, durvalumab, and paclitaxel combination in BEGONIA; however, this confirmed ORR was not maintained in the subsequent analysis following study expansion of CO40151 (32).

In mTNBC, CD73 is frequently upregulated in the tumor microenvironment, and extracellular adenosine is believed to mediate the immunosuppressive effects of both myeloid-derived suppressor cells and regulatory T cells, among others (33). Oleclumab (MEDI9447) is a human mAb that selectively targets and inhibits CD73, thus preventing the catalysis of adenosine monophosphate to adenosine and organic phosphate (34, 35). The enzymatic blockade of CD73 may therefore lead to increased antitumor activity. Results from phase I and II trials demonstrated the targeted activity of oleclumab in combination with durvalumab in patients with solid tumors (36, 37). Furthermore, preclinical data indicated that targeted blockade of CD73 could enhance a chemotherapy-mediated antitumor immune response and prolong survival in mouse models of TNBC (22).

Together, these combinations integrate immune activation, oncogenic signaling inhibition, and microenvironment modulation to maximize antitumor efficacy. Thus, evaluation of their combination as a treatment strategy was warranted. BEGONIA was a phase Ib/II, multiarm, platform study that evaluated the safety and efficacy of durvalumab combined with novel anticancer therapies, with or without paclitaxel, as first-line treatment in patients with locally advanced, unresectable or mTNBC (Supplementary Fig. S1). Results from arm 6, which evaluated durvalumab plus trastuzumab deruxtecan, arm 7, which evaluated durvalumab plus datopotamab deruxtecan, and arm 8, which evaluated durvalumab plus datopotamab deruxtecan in patients with PD-L1–positive tumors, have been previously reported (38, 39). Here, we report results from 3 chemotherapy combination arms: durvalumab in combination with paclitaxel (arm 1) and durvalumab plus paclitaxel in combination with capivasertib (arm 2) or oleclumab (arm 5).

## Patients and Methods

### Patients

Female patients ≥18 years of age with untreated, unresectable, locally advanced or metastatic stage IV TNBC, at least 1 unirradiated target lesion per Response Evaluation Criteria in Solid Tumors (RECIST) v1.1, an Eastern Cooperative Oncology Group performance status of 0 or 1, and adequate organ and marrow function were enrolled. TNBC was defined as ER, PR, and HER2 negative according to the American Society of Clinical Oncology/College of American Pathologists testing guidelines (40, 41). Prior treatment for stage I to III TNBC was permitted if ≥6 months had elapsed from the completion of treatment to the first documented distant recurrence and ≥12 months had elapsed since the last administration of taxanes. Exclusion criteria included active or prior autoimmune or inflammatory disorders or prior immunotherapy use for all arms and prior treatment with PI3K, AKT, or mTOR inhibitors for the capivasertib-combination arm only. Full eligibility criteria are in the Study Protocol provided as Supplementary Material.

### Study design

BEGONIA (NCT03742102) is a multicenter, multiarm, open-label, phase Ib/II platform study initiated to concurrently evaluate first-line durvalumab combined with novel anticancer therapies with or without paclitaxel for mTNBC using a Simon two-stage design (Supplementary Fig. S1; ref. 42). The primary objective of part 1 was to assess the safety and tolerability of durvalumab combinations. The efficacy of durvalumab combinations was a secondary objective in part 1 and a primary objective in part 2.

In part 1 of the study, the first 20 evaluable patients were enrolled into the durvalumab plus paclitaxel arm (arm 1) and monitored for toxicity. Subsequently enrolled patients were randomized into an open, novel treatment combination arm. For each arm, the first 6 patients were monitored for dose-limiting toxicities (DLT), with additional patients enrolled if treatment was tolerated (around 30 total evaluable patients). All patients either completed at least 2 on-treatment response evaluations or discontinued treatment. If the ORR was at least 57% in a given novel treatment combination arm, based on confirmed responses from 30 patients, the arm was permitted to proceed to part 2 expansion (with approximately 27 additional evaluable patients enrolled).

The study was conducted at multiple centers in the United States, Canada, Poland, the United Kingdom, the Republic of Korea, and Taiwan in accordance with the International Council for Harmonization Good Clinical Practice guidelines, the Declaration of Helsinki, and all applicable regulations. The protocol was reviewed and approved by an institutional review board or independent ethics committee at each site. Written informed consent was obtained from all participants.

### Treatment

In the 3 treatment arms presented here, durvalumab was administered at 1,500 mg intravenously every 4 weeks. In the durvalumab plus paclitaxel and the oleclumab-combination arms, paclitaxel was administered at 90 mg/m^2^ intravenously on days 1, 8, and 15, with 1 week off, in 4-week cycles. In the capivasertib-combination arm, patients were initially assigned to receive paclitaxel 90 mg/m^2^ intravenously on the same schedule; however, this was subsequently amended to 80 mg/m^2^ to align with the ongoing phase III CAPItello-290 trial (NCT03997123; ref. 30). Capivasertib was given orally in 4-week cycles, consisting of a 400 mg dose twice daily on days 2 to 5, 9 to 12, and 16 to 19. Oleclumab was given at 3,000 mg intravenously on days 1 and 15 of the first 2 cycles and then once every 4 weeks thereafter. Dose reductions were not permitted for durvalumab or oleclumab but were permitted for capivasertib according to the protocol-specified schema. Paclitaxel-related toxicity was managed per local guidelines and standard clinical practice.

### Endpoints, assessments, and biomarkers

Safety was assessed through physical examinations, vital signs, clinical laboratory tests, electrocardiograms, and an echocardiogram or multigated acquisition scan. A safety review committee evaluated the safety and tolerability of each novel treatment combination during the run-in period and continually throughout the study. Adverse events (AE) were reported per the National Cancer Institute Common Terminology Criteria for Adverse Events grading scale version 4.03. Immune-mediated AEs were defined as AEs of special interest consistent with an immune-mediated mechanism of action with no clear alternate etiology and which required the use of systemic corticosteroids, other immunosuppressants, or endocrine therapy to manage.

Tumors were investigator assessed by computed tomography or magnetic resonance imaging every 8 weeks for the first 48 weeks and then every 12 weeks per RECIST v1.1 for the 3 arms. ORR was a key secondary endpoint for part 1 and the primary endpoint for part 2. PFS, duration of response (DOR), and OS were secondary endpoints in both study parts. Tumor biomarker analysis was conducted retrospectively as an exploratory objective.

PD-L1 expression was assessed centrally and retrospectively by immunohistochemistry (IHC) using the VENTANA PD-L1 (SP263) Assay (Roche Diagnostics, cat. #790-4905, RRID: AB_2819099). Expression was defined as the percentage of the tumor area populated by tumor cells with membranous PD-L1 staining or immune cells with membranous, cytoplasmic, or punctuate PD-L1 staining at any intensity [tumor area positivity (TAP) score]. A tumor sample was considered PD-L1 positive if the TAP score was ≥10% and PD-L1 negative if the TAP score was <10%.

*PIK3CA/AKT1/PTEN* alterations were assessed centrally and retrospectively via next-generation sequencing of tumor tissue using the FoundationOne CDx assay (Foundation Medicine, Inc.) or circulating tumor (ct)DNA using the GuardantOMNI assay (Guardant Health Inc.). A tumor sample was considered positive if a qualifying genetic alteration (43) in *PIK3CA*, *AKT1*, or *PTEN* was detected by either assay.

CD73 expression was assessed centrally and retrospectively via IHC using the rabbit mAb EPR6115 (Abcam, cat. #ab124725, RRID: AB_10976033). Expression was defined as the percentage of stromal immune cells demonstrating detectable CD73. A tumor sample was considered CD73^+^ if expression was ≥1% and CD73^− ^if it was <1%.

### Statistical analysis

For part 1, a sample size of 20 patients in the durvalumab plus paclitaxel arm and 30 patients in the novel treatment combination arms was considered sufficient to evaluate the safety of these treatments. This sample size also allowed evaluation of the targeted ORR improvement from 55% to 75% with 94% power and 5% α level for Simon two-stage assessment of each novel treatment combination arm. The study arms were permitted to proceed to part 2 expansion if ORR was at least 57% (based on confirmed responses from 30 patients) in part 1.

Descriptive statistics were used for baseline patient and disease characteristics and safety assessments. For each arm, the safety analysis was performed in patients who received at least one dose of study treatment (safety analysis set). ORR was defined as the percentage of patients with at least 1 confirmed CR or partial response of all treated patients with measurable disease at baseline who completed at least 2 on-treatment disease assessments (response-evaluable analysis set). PFS was measured from the date of the first dose of study drug until the date of progression or death. DOR was measured from the date of the first documented confirmed response to the date of progression or death. OS was measured from the date of the first dose of study drug until the date of death. PFS, DOR, and OS were measured for all patients who were assigned to treatment and received at least 1 dose of study treatment (intent-to-treat set). ORR exact 95% CIs were calculated using the Clopper–Pearson method. Time-to-event endpoints were calculated using the Kaplan–Meier method. Statistical analyses were done using Statistical Analysis System (SAS) version 9.4 (RRID: SCR_008567).

## Results

### Patients

Between January 3, 2019, and October 13, 2020, 87 female patients were enrolled across the 3 treatment arms. Patients received either durvalumab plus paclitaxel (*n* = 23) or were randomly assigned to receive durvalumab plus paclitaxel in combination with capivasertib (*n* = 31; capivasertib combination) or durvalumab plus paclitaxel in combination with oleclumab (*n* = 33; oleclumab combination; Supplementary Fig. S2). Most patients were ≥50 years of age, had received treatment for early-stage breast cancer, and had disease spread to visceral organs and/or the lymph nodes (Table 1). Study representativeness is shown in Supplementary Table S1. Of those with PD-L1–evaluable samples, 4 of 21 (19%), 4 of 29 (13.8%), and 6 of 33 (18.2%) were PD-L1 positive in the 3 arms, respectively. In the capivasertib-combination arm, 28 (90.3%) patients had evaluable aggregated ctDNA plasma and tumor tissue samples for *PIK3CA/AKT1/PTEN* alterations, 8 (28.6%) of whom had tumors that tested positive, whereas in the oleclumab-combination arm, 24 (72.7%) patients had evaluable tumor tissue for CD73 expression and 19 (79.2%) tested positive.

**Table 1.** Baseline patient demographics and disease characteristics.

Parameter	Durvalumab + paclitaxel (*N* = 23)	Durvalumab + paclitaxel + capivasertib (*N* = 31)	Durvalumab + paclitaxel + oleclumab (*N* = 33)
Age, median (range), years	52 (30–62)	51 (32–71)	54 (33–72)
Age group, years, *n* (%)	​	​	​
<65	23 (100)	26 (83.9)	27 (81.8)
≥65 to <75	0	5 (16.1)	6 (18.2)
Race, *n* (%)	​	​	​
Asian	17 (73.9)	16 (51.6)	17 (51.5)
Black or African American	0	0	1 (3)
White	5 (21.7)	15 (48.4)	15 (45.5)
Other	1 (4.3)	0	0
No prior treatment, *n* (%)	6 (26.1)	14 (45.2)	13 (39.4)
Treatment for prior breast cancer, *n* (%)	​	​	​
Radiotherapy	15 (65.2)	15 (48.4)	19 (57.6)
Cytotoxic chemotherapy	17 (73.9)	16 (51.6)	20 (60.6)
Nitrogen mustard analogues	15 (65.2)	16 (51.6)	18 (54.5)
Anthracycline and related substances	15 (65.2)	15 (48.4)	17 (51.5)
Taxanes	14 (60.9)	13 (41.9)	18 (54.5)
Hormonal therapy	1 (4.3)	2 (6.5)	3 (9.1)
Targeted therapy^a^	1 (4.3)	0	3 (9.1)
Visceral metastases^b^, *n* (%)	13 (56.5)	19 (61.3)	21 (63.6)
Lymph node metastases, *n* (%)	16 (69.6)	19 (61.3)	18 (54.5)
Eastern Cooperative Oncology Group performance status, *n* (%)	​	​	​
0	17 (73.9)	17 (54.8)	20 (60.6)
1	6 (26.1)	14 (45.2)	13 (39.4)
PD-L1 expression^c^, *n* (%)	​	​	​
Positive (PD-L1 TAP ≥10%)	4 (17.4)	4 (12.9)	6 (18.2)
Negative (PD-L1 TAP <10%)	17 (73.9)	25 (80.6)	27 (81.8)
Missing score	2 (8.7)	2 (6.5)	0
*PIK3CA/AKT1/PTEN* alterations^d^, *n* (%)	​	​	​
Yes	3 (13)	8 (25.8)	—
No	11 (47.8)	20 (64.5)	—
Missing score	9 (39.1)	3 (9.7)	—
CD73 expression^e^, *n* (%)	​	​	​
Positive (≥1%)	10 (43.5)	—	19 (57.6)
Negative (<1%)	7 (30.4)	—	5 (15.2)
Missing score	6 (26.1)	—	9 (27.3)

a In the durvalumab and paclitaxel arm, 1 patient received pertuzumab and trastuzumab. In the oleclumab-combination arm, 2 patients received trastuzumab and 1 patient received pertuzumab and trastuzumab.

b Visceral metastases include liver/hepatic and/or respiratory metastases.

c Assessed via IHC using the VENTANA PD-L1 (SP263) Assay (Roche Diagnostics). Expression was defined as the percentage of the tumor area populated by tumor cells with membranous staining for PD-L1 or immune cells with membranous, cytoplasmic, or punctate PD-L1 staining at any intensity (TAP score). A TAP score ≥10% was considered positive, whereas a TAP score <10% was considered negative.

d Number of patients with aggregated plasma ctDNA and tissue *PIK3CA/AKT1/PTEN* alterations. A patient was considered positive if a qualifying genetic alteration in *PIK3CA*, *AKT1*, or *PTEN* was detected by either assay.

e Assessed via IHC. Expression was defined by the percentage of stromal immune cells with CD73 detected. A sample was considered positive if expression was ≥1% and negative if it was <1%.

### Exposure

Capivasertib dose reductions were reported for 14 (45.2%) patients. Paclitaxel dose reductions were reported for 14 (60.9%) patients in the durvalumab plus paclitaxel arm, 15 (48.4%) in the capivasertib-combination arm, and 14 (42.4%) in the oleclumab-combination arm (Supplementary Table S2). Among patients who had discontinued study treatment, 17 of 21 (81%), 22 of 28 (78.6%), and 26 of 31 (83.9%), respectively, received subsequent anticancer therapy, with disease progression being the most common reason for discontinuation (Supplementary Fig. S2). Subsequent therapy was initiated after progression in 17 (81%), 21 (75%), and 23 (74.2%) patients in the durvalumab plus paclitaxel, capivasertib-combination, and oleclumab-combination arms, respectively. Subsequent therapy was initiated before progression in 1 (3.2%) patient in the oleclumab-combination arm and in the absence of progression in 1 (3.6%) patient in the capivasertib-combination arm and 2 (6.5%) patients in the oleclumab-combination arm.

### Safety

No DLTs were observed with the capivasertib or oleclumab combinations during the safety run-in. An overview of safety across arms is provided in Table 2. Maximum grade 3 or 4 AEs occurred in 10 (43.5%) patients in the durvalumab plus paclitaxel arm, 25 (80.6%) in the capivasertib-combination arm, and 8 (24.2%) in the oleclumab-combination arm. There were no AEs with outcome of death in any arm.

**Table 2.** Summary of AEs.

Parameter, *n* (%)	Durvalumab + paclitaxel (*N* = 23)	Durvalumab + paclitaxel + capivasertib (*N* = 31)	Durvalumab + paclitaxel + oleclumab (*N* = 33)
Any AE	22 (95.7)	31 (100)	32 (97)
Any AE possibly related to treatment	22 (95.7)	31 (100)	32 (97)
Possibly related to durvalumab only	10 (43.5)	13 (41.9)	10 (30.3)
Possibly related to novel oncology therapy only	N/A	26 (83.9)	3 (9.1)
Any AE of maximum CTCAE grade 3 or 4	10 (43.5)	25 (80.6)	8 (24.2)
Possibly related to treatment	10 (43.5)	24 (77.4)	7 (21.2)
Possibly related to durvalumab only	3 (13)	3 (9.7)	0
Possibly related to novel oncology therapy only	N/A	12 (38.7)	0
Any AE with outcome of death	0	0	0
Any SAE	1 (4.3)	11 (35.5)	4 (12.1)
Possibly related to treatment	1 (4.3)	7 (22.6)	1 (3)
Possibly related to durvalumab only	1 (4.3)	3 (9.7)	0
Possibly related to novel oncology therapy only	0	1 (3.2)	0
Any AE leading to discontinuation of any study treatment	4 (17.4)	7 (22.6)	6 (18.2)
Any AE leading to discontinuation of durvalumab	1 (4.3)	1 (3.2)	0
Any AE leading to discontinuation of paclitaxel	3 (13)	6 (19.4)	6 (18.2)
Any AE leading to discontinuation of novel oncology therapy	N/A	1 (3.2)	0
Any AE leading to dose interruption of any study treatment	13 (56.5)	28 (90.3)	17 (51.5)
Any AE leading to dose reduction of any study treatment^a^	12 (52.2)	20 (64.5)	10 (30.3)
Any AE leading to dose reduction of paclitaxel^b^	12 (52.2)	12 (38.7)	10 (30.3)
Any AE leading to dose reduction of novel oncology therapy^b^	N/A	11 (35.5)	1 (3)^c^
Any imAE^d^	5 (21.7)	15 (48.4)	9 (27.3)

Abbreviations: CTCAE, Common Terminology Criteria for Adverse Events; imAE, immune-mediated adverse event; N/A, not applicable; SAE, serious adverse event.

a Dose reduction was not allowed for durvalumab and oleclumab.

b Related to given treatment, as assessed by the investigator.

c Dose reduction for oleclumab was not reflected on exposure.

d imAEs were defined as AEs of special interest that were consistent with an immune-mediated mechanism of action with no clear alternative cause and resulted in the use of systemic glucocorticoids, other immunosuppressants, or endocrine therapy.

The most common AEs were peripheral neuropathy (69.6%), alopecia (60.9%), and nausea (47.8%) with durvalumab plus paclitaxel; diarrhea (67.7%), rash (58.1%), and peripheral neuropathy (41.9%) with capivasertib combination; and peripheral neuropathy (63.6%), alopecia (48.5%), and headache (36.4%) with oleclumab combination (Table 3). The most common maximum grade 3 or 4 AEs were decreased neutrophil count (17.4%) for patients treated with durvalumab plus paclitaxel, diarrhea (19.4%) for capivasertib combination, and peripheral neuropathy (9.1%) for oleclumab combination.

**Table 3.** Most common AEs (≥20% of patients in any arm).

AEs by preferred term, *n* (%)	Durvalumab + paclitaxel (*N* = 23)		Durvalumab + paclitaxel + capivasertib (*N* = 31)		Durvalumab + paclitaxel + oleclumab (*N* = 33)	
	Any grade	Maximum grade 3 or 4	Any grade	Maximum grade 3 or 4	Any grade	Maximum grade 3 or 4
Any AE	22 (95.7)	10 (43.5)	31 (100)	25 (80.6)	32 (97)	8 (24.2)
Peripheral neuropathy^a^	16 (69.6)	1 (4.3)	13 (41.9)	2 (6.5)	21 (63.6)	3 (9.1)
Alopecia	14 (60.9)	—	11 (35.5)	—	16 (48.5)	—
Nausea	11 (47.8)	—	8 (25.8)	—	11 (33.3)	—
Fatigue	9 (39.1)	—	10 (32.3)	—	6 (18.2)	—
Rash	9 (39.1)	1 (4.3)	18 (58.1)	4 (12.9)	9 (27.3)	—
Neutrophil count decreased	7 (30.4)	4 (17.4)	4 (12.9)	2 (6.5)	2 (6.1)	1 (3)
Edema peripheral	7 (30.4)	—	1 (3.2)	—	6 (18.2)	—
Hypothyroidism	6 (26.1)	—	1 (3.2)	—	7 (21.2)	—
Myalgia	6 (26.1)	—	3 (9.7)	—	8 (24.2)	—
ALT increased	5 (21.7)	1 (4.3)	5 (16.1)	1 (3.2)	2 (6.1)	1 (3)
Nail discoloration	5 (21.7)	—	—	—	—	—
Pruritus	5 (21.7)	—	10 (32.3)	—	6 (18.2)	—
Pyrexia	4 (17.4)	—	8 (25.8)	—	2 (6.1)	—
Cough	3 (13)	—	8 (25.8)	—	7 (21.2)	—
Diarrhea	3 (13)	—	21 (67.7)	6 (19.4)	11 (33.3)	—
Neutropenia	3 (13)	1 (4.3)	8 (25.8)	5 (16.1)	—	—
Urinary tract infection	3 (13)	—	5 (16.1)	—	2 (6.1)	—
Anemia	2 (8.7)	—	7 (22.6)	2 (6.5)	4 (12.1)	—
Headache	2 (8.7)	—	5 (16.1)	—	12 (36.4)	—
Hyperglycemia	—	—	10 (32.3)	4 (12.9)	1 (3)	1 (3)

Abbreviation: ALT, alanine aminotransferase.

AEs were reported per the National Cancer Institute Common Terminology Criteria for Adverse Events grading scale.

Patients with multiple AEs are counted once for each preferred AE term. AEs with an onset date on/after the date of first dose; AEs with an onset date prior to dosing which worsen after dosing; AEs occurring up to 90 days following the date of last dose or up to first subsequent therapy, whichever occurs first, are reported.

a Includes “peripheral sensory neuropathy” and “neuropathy peripheral” preferred terms.

The most common AEs possibly related to durvalumab were rash (26.1%), hypothyroidism (26.1%), and fatigue (17.4%) in the durvalumab and paclitaxel arm; rash (25.8%), pruritus (22.6%), and fatigue (19.4%) in the capivasertib-combination arm; and hypothyroidism (21.2%), rash (12.1%), and fatigue (15.2%) in the oleclumab-combination arm (Supplementary Table S3). The most common AEs possibly related to capivasertib were diarrhea (64.5%), rash (48.4%), and hyperglycemia (29%; Supplementary Table S4), whereas the most common AEs possibly related to oleclumab were fatigue (15.2%) and nausea (12.1%; Supplementary Table S5).

Serious AEs were experienced by 1 (4.3%) patient receiving durvalumab plus paclitaxel, 11 (35.5%) receiving the capivasertib combination, and 4 (12.1%) receiving the oleclumab combination (Table 2). Pyrexia and febrile neutropenia were the most frequently reported serious AEs (6.5% each), both being reported only in the capivasertib-combination arm. AEs leading to discontinuation of at least 1 study treatment occurred in 4 (17.4%), 7 (22.6%), and 6 (18.2%) patients in the three arms, respectively (Table 2). Peripheral neuropathy was the most frequent AE leading to discontinuation of any study treatment [3 (9.7%) with capivasertib combination and 4 (12.1%) with oleclumab combination]. Immune-mediated AEs were reported in 5 (21.7%) patients in the durvalumab plus paclitaxel arm, 15 (48.4%) in the capivasertib arm, and 9 (27.3%) in the oleclumab arm (Table 2); the most common AEs were hypothyroidism in the durvalumab plus paclitaxel (21.7%) and oleclumab-combination (21.2%) arms and rash (25.8%) in the capivasertib-combination arm.

### Antitumor activity

**Figure 1.**
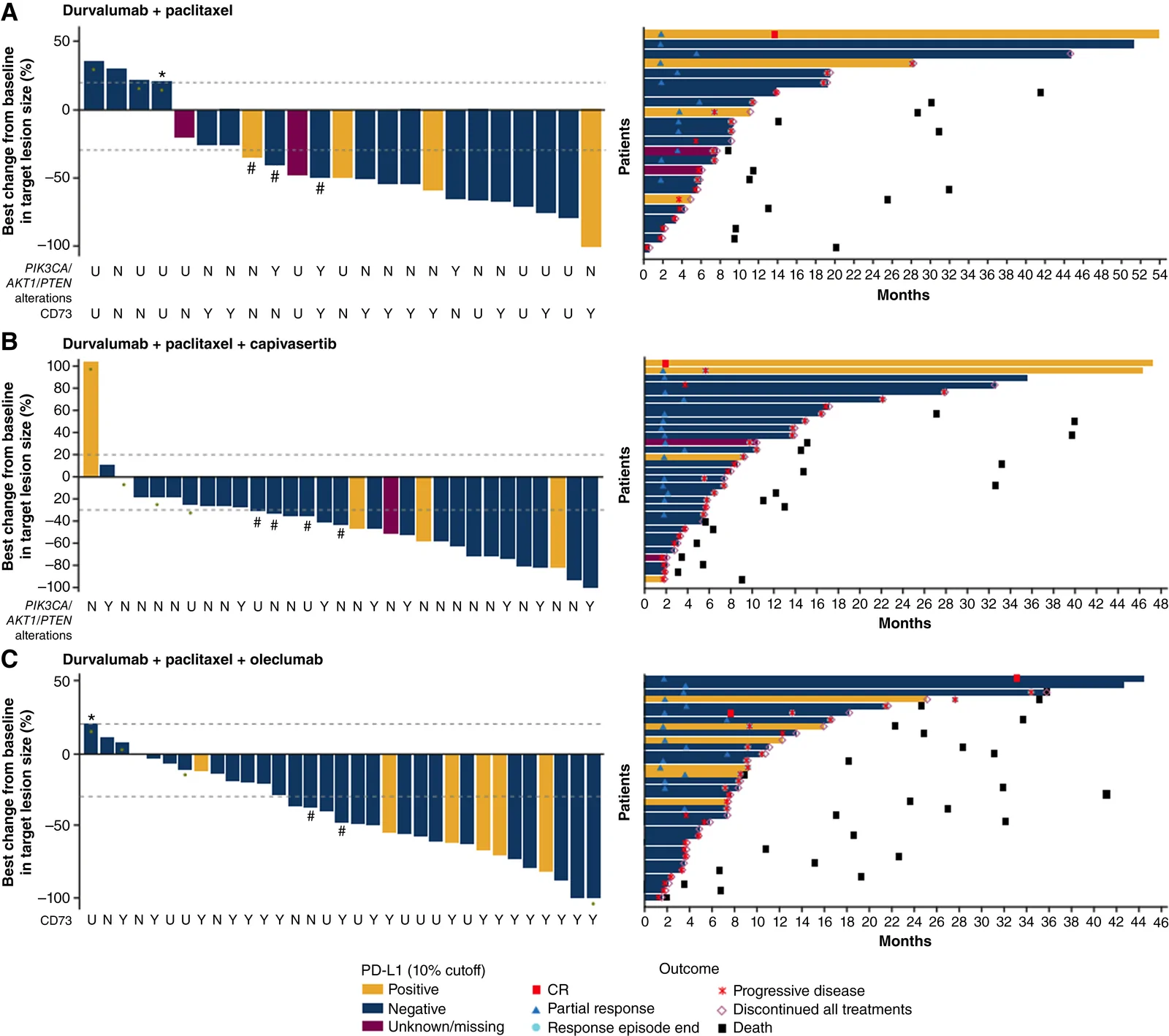
**A–C, **Best change from baseline in target lesion size (left panels) and DOR (right panels). Left, dotted lines indicate thresholds for partial response (−30%) and progressive disease (20%). One patient who received the oleclumab combination had a CR in their target lesion but experienced progressive disease in a nontarget lesion, with an overall response of progressive disease. *, If the best percentage change from baseline of target lesions was not calculated because of progression, withdrawal, or death, the value was imputed at +20%; 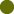, patients with progressive disease as best overall response; #, unconfirmed response. Right, each bar represents an individual patient. Partial response and CR to treatment as well as disease progression are shown as symbols within the colored bars; stable disease is not represented. The length of each bar indicates the duration of treatment. PD-L1 expression was assessed with the VENTANA PD-L1 (SP263) Assay (Roche Diagnostics). Expression was defined as the percentage of the tumor area populated by tumor cells with membranous staining for PD-L1 or immune cells with membranous, cytoplasmic, or punctate PD-L1 staining at any intensity (TAP score). A TAP score ≥10% was considered positive, whereas a TAP score <10% was considered negative. N, no; U, unknown; Y, yes.

The median follow-up period in the durvalumab plus paclitaxel, capivasertib-combination, and oleclumab-combination arms was 51.2 months (range, 10–54), 36.2 months (3–49), and 43.3 months (10–48), respectively. All 87 patients had measurable disease at baseline and completed at least 2 on-treatment disease assessments. The confirmed ORR was similar among the 3 treatment arms: 56.5% (13/23; 95% CI, 34.5–76.8) in the durvalumab plus paclitaxel arm, 54.8% (17/31; 36–72.7) in the capivasertib-combination arm, and 51.5% (17/33; 33.5–69.2) in the oleclumab combination arm (Table 4). The ORR for both the capivasertib- and oleclumab-combination arms did not meet the prespecified criteria for part 2 expansion. CRs were observed in 1 (4.3%) patient in the durvalumab plus paclitaxel arm, 1 (3.2%) in the capivasertib-combination arm, and 2 (6.1%) in the oleclumab-combination arm (Fig. 1). The median PFS was 7.3 months (95% CI, 5.4–11.3) with durvalumab plus paclitaxel, 6.4 months (5.4–10.3) with capivasertib combination, and 8.3 months (4.8–9.3) with oleclumab combination (Table 4; Supplementary Fig. S3). The median DOR was 5.6 months (95% CI, 3.8–26.1) with durvalumab plus paclitaxel, 7.9 months (4–14.6) with capivasertib combination, and 7.8 months (5.4–17.8) with oleclumab combination (Fig. 1; Table 4). The median OS was 30 months (95% CI, 13–not calculable) with durvalumab plus paclitaxel, 32.6 months (12.9–40) with capivasertib combination, and 24.8 months (18.6–31.9) with oleclumab combination (Table 4; Supplementary Fig. S4).

**Table 4.** Response and survival outcomes.

Outcome	Durvalumab + paclitaxel (*N* = 23)	Durvalumab + paclitaxel + capivasertib (*N* = 31)	Durvalumab + paclitaxel + oleclumab (*N* = 33)
Confirmed ORR, *n* (%)	13 (56.5)	17 (54.8)	17 (51.5)
95% CI	34.5–76.8	36–72.7	33.5–69.2
CR	1 (4.3)	1 (3.2)	2 (6.1)
PR	12 (52.2)	16 (51.6)	15 (45.5)
SD^a^	7 (30.4)	10 (32.3)	12 (36.4)
PD	3 (13)	4 (12.9)	4 (12.1)
Median PFS (95% CI), months	7.3 (5.4–11.3)	6.4 (5.4–10.3)	8.3 (4.8–9.3)
Median DOR (95% CI), months	5.6 (3.8–26.1)	7.9 (4–14.6)	7.8 (5.4–17.8)
Median OS (95% CI), months	30 (13–NC)	32.6 (12.9–40)	24.8 (18.6–31.9)

Abbreviations: NC, not calculable; PD, progressive disease; PR, partial response; SD, stable disease.

ORR exact 95% CIs were calculated using the Clopper–Pearson method. The median DOR, PFS, and OS were calculated using the Kaplan–Meier method.

a SD was recorded ≥7 weeks after treatment assignment.

Although the number of patients with PD-L1–positive tumors was small in each arm, confirmed ORR and PFS were numerically higher in this population than in patients with PD-L1–negative tumors (Table 5). The respective confirmed ORR was 75% (3/4) versus 52.9% (9/17) in the durvalumab plus paclitaxel arm, 75% (3/4) versus 52% (13/25) in the capivasertib-combination arm, and 83.3% (5/6) versus 44.4% (12/27) in the oleclumab-combination arm. The respective median PFS was 17.5 versus 7.3 months with durvalumab plus paclitaxel, 7.4 versus 6.4 with capivasertib combination, and 9.2 versus 7.3 with oleclumab combination. Differences in efficacy outcomes were also observed in a biomarker analysis of patients with tumors harboring *PIK3CA/AKT1/PTEN* alterations, suggesting numerically lower ORR and shorter PFS in patients with altered compared with nonaltered tumors treated with durvalumab plus paclitaxel, and numerically higher ORR, PFS, and DOR in patients with altered compared with nonaltered tumors in the capivasertib-combination arm (Supplementary Table S6). However, the small number of patients in these groups limits data interpretation. In the durvalumab plus paclitaxel and the oleclumab-combination arms, patients with positive CD73 had numerically higher ORR and longer PFS and DOR than those with negative CD73 (Supplementary Table S7).

**Table 5.** Response and survival outcomes by PD-L1 expression.

Outcome^a^	Durvalumab + paclitaxel (*N* = 4)	Durvalumab + paclitaxel + capivasertib (*N* = 4)	Durvalumab + paclitaxel + oleclumab (*N* = 6)
PD-L1 positive			
Confirmed ORR, *n* (%)	3 (75)	3 (75)	5 (83.3)
Median PFS (95% CI), months	17.5 (3.6–NC)	7.4 (1.7–NC)	9.2 (7.3–NC)
Median DOR (95% CI), months	26.1 (3.7–NC)	7.4 (4–NC)	7.8 (5–NC)
Median OS (95% CI), months	NC (25.5–NC)	NC (9–NC)	29.4 (8.9–NC)
**PD-L1 negative**	***N* = 17**	***N* = 25**	***N* = 27**
Confirmed ORR, *n* (%)	9 (52.9)	13 (52)	12 (44.4)
Median PFS (95% CI), months	7.3 (3.1–13.8)	6.4 (5.4–13.7)	7.3 (3.6–10.4)
Median DOR (95% CI), months	5.6 (3.8–NC)	11.9 (3.9–14.6)	8.3 (3.7–30.9)
Median OS (95% CI), months	30.8 (13–NC)	32.6 (12.2–40)	24.8 (18.2–31.9)

Abbreviation: NC, not calculable.

PD-L1 expression was assessed with the VENTANA PD-L1 (SP263) Assay (Roche Diagnostics). Expression was defined as the percentage of the tumor area populated by tumor cells with membranous staining for PD-L1 or immune cells with membranous, cytoplasmic, or punctate PD-L1 staining at any intensity (TAP score). A TAP score ≥10% was considered positive, whereas a TAP score <10% was considered negative.

The median DOR, PFS, and OS were calculated using the Kaplan–Meier method.

a Two patients in the durvalumab plus paclitaxel arm and 2 patients in the capivasertib-combination arm had missing PD-L1 expression data.

## Discussion

In the BEGONIA trial, durvalumab plus paclitaxel demonstrated expected efficacy and safety for an ICI–chemotherapy combination treatment in locally advanced, unresectable/mTNBC. The safety profiles observed were consistent with the known profiles of each agent, with no unexpected toxicities observed. Although BEGONIA was not designed for direct comparison between arms, the addition of capivasertib or oleclumab to durvalumab plus paclitaxel in a biomarker-unselected patient population showed no substantial additional benefit in the response rate or DOR. Responses occurred for 51.5% to 56.5% of patients across all treatment arms, aligning with those reported for other ICI–chemotherapy combinations (32).

In BEGONIA, patients with PD-L1–positive disease demonstrated a higher response rate (75%–83.3% vs. 44.4%–52.9%) and better survival outcomes (median OS, 29.4 months–not calculable vs. 24.8–32.6 months) across all arms versus patients who had PD-L1–negative disease. Although these findings are limited by the small number of PD-L1–positive patients, they are consistent with prior evidence that clinical efficacy of ICI plus chemotherapy as shown in larger phase III studies is greater in patients with PD-L1–positive disease versus PD-L1–negative disease (6, 12, 44). Notably, pembrolizumab and atezolizumab are currently approved only for use in patients with PD-L1–positive tumors in the metastatic setting (45, 46). In the KEYNOTE-355 study, for patients with PD-L1 combined positive score ≥10 versus <10, the ORR was 53.2% versus 33.2%, the median PFS was 9.7 versus 5.8 months, and the median OS was 23 versus 14.7 months. Similarly, in the IMpassion130 study, for patients with PD-L1 TAP ≥1% versus <1%, the ORR was 58.9% versus 53.9%, the median PFS was 7.5 versus 5.6 months, and the median OS was 25 versus 19.7 months.

The combination of capivasertib with durvalumab plus paclitaxel in BEGONIA elicited a higher response rate and longer OS but comparable PFS with those reported previously for first-line capivasertib plus paclitaxel in patients with mTNBC (28, 29). For the overall population in this arm, we report an ORR of 54.8%, a median PFS of 6.4 months, and a median OS of 32.6 months. The efficacy results in the PAKT trial were an ORR of 34.8%, a median PFS of 5.9 months, and a median OS of 19.1 months (28, 29). Although limited by the small number of patients included in these analyses, we also observed a numerically higher response rate, PFS, and DOR for patients with versus without *PIK3CA/AKT1/PTEN*-altered tumors in the capivasertib-combination arm. This aligns with findings from studies investigating first-line combination of ipatasertib, atezolizumab, and a taxane for locally advanced or mTNBC, in which patients with PFS >10 months had disease characterized by *NF1*, *CCND3*, and *PIK3CA* alterations and increased immune pathway activity (32). In contrast, patients with *PIK3CA/AKT1/PTEN*-altered tumors treated with durvalumab plus paclitaxel showed numerically lower response rates and shorter PFS and DOR versus those with *PIK3CA/AKT1/PTEN*-nonaltered tumors. In terms of safety, patients treated with the combination of capivasertib with durvalumab and paclitaxel had higher rates of grade 3 or 4 AEs, serious AEs, and AEs leading to dose interruptions and reductions compared with those who received durvalumab plus paclitaxel. However, these AEs were manageable, consistent with those previously reported for capivasertib. Hyperglycemia and rash, common with the class of AKT inhibitors, were observed but did not lead to discontinuation of treatment.

In the oleclumab-combination arm, we observed an ORR of 51.5%, a median PFS of 8.3 months, and a median OS of 24.8 months, demonstrating no benefit for the addition of oleclumab to durvalumab plus paclitaxel in a biomarker-unselected patient population. Previous studies have reported an association between CD73 expression and poor prognosis in TNBC (20, 22). In our analysis, this association was observed in both durvalumab plus paclitaxel and oleclumab-combination arms. Despite the limited sample size in these analyses, we observed a numerically higher response rate, PFS, and DOR for patients with positive CD73 versus those with negative CD73 in both the durvalumab plus paclitaxel and oleclumab-combination arms. These results align with the phase II SYNERGY study in patients with untreated, advanced, or mTNBC, who were randomized to receive durvalumab plus paclitaxel and carboplatin with or without oleclumab. In this study, there was no statistically significant increase in the clinical benefit rate with versus without oleclumab (43% vs. 44%, *P* = 0.61). The median PFS was numerically shorter with versus without oleclumab (5.9 vs. 7 months; *P* = 0.90), whereas the median OS showed numerical improvement (25.1 vs. 19.3 months; *P* = 0.62); however, these differences were not statistically significant (47). Moreover, a numerical difference in the clinical benefit rate was observed in SYNERGY when patients were stratified according to CD73 expression, with higher rates observed in those with positive status, although the difference was not statistically significant. In terms of safety in the BEGONIA oleclumab-combination arm, AEs were consistent with those previously reported for oleclumab (47).

The BEGONIA trial highlights the value of a platform study design for assessing the safety and preliminary efficacy of several novel treatment combinations within a single protocol in the first-line mTNBC setting. Although the addition of oleclumab or capivasertib to durvalumab plus paclitaxel did not yield improved outcomes compared with the durvalumab plus paclitaxel combination, the data presented here are in line with those observed for other ICIs in combination with chemotherapy. These findings demonstrate that durvalumab plus paclitaxel has a comparable efficacy and safety with other PD-1/PD-L1 inhibitors and support the continued investigation and potential use of durvalumab-based regimens in the first-line treatment of TNBC.

The BEGONIA trial was limited by the small population enrolled to each arm and that patients were not prospectively enriched for specific biomarkers. Randomization was not stratified, and arms became open to enrollment at different times, which may have led to imbalances in the characteristics between the analysis populations. Asian participants were overrepresented in the durvalumab plus paclitaxel arm. There were a lower proportion of patients with *de novo* mTNBC in the durvalumab plus paclitaxel arm compared with the other arms. Moreover, BEGONIA had a predominantly PD-L1–negative patient population; the low prevalence of patients with PD-L1–positive tumors was likely due to the availability of new standard-of-care ICIs for these patients at the time of enrollment. Finally, the limited availability of evaluable tumor tissue hampered the interpretation of potential benefit in biomarker-selected subpopulations.

In conclusion, first-line treatment with durvalumab plus paclitaxel in patients with locally advanced, unresectable or mTNBC demonstrated expected efficacy and safety for an ICI plus chemotherapy combination. The addition of capivasertib or oleclumab to durvalumab plus paclitaxel in this biomarker-unselected patient population showed no apparent additional benefit, consistent with previous studies evaluating similar therapeutic combinations.

## Supplementary Material

Supplementary Table S1 Representativeness of study participants

Supplementary Table S2 Summary of exposure, treatment delays, and dose reductions

Supplementary Table S3 Most common AEs (in ≥5% of patients in any arm) possibly related to durvalumab

Supplementary Table S4 Most common AEs (in ≥5% of patients) possibly related to capivasertib

Supplementary Table S5 Most common AEs (in ≥5% of patients) possibly related to oleclumab

Supplementary Table S6 Response and survival outcome per detection of PIK3CA/AKT1/PTEN alterations

Supplementary Table S7 Response and survival outcome per CD73 expression status

Supplementary Figure S1 Study design

Supplementary Figure S2 Patient disposition in three arms from BEGONIA

Supplementary Figure S3 Kaplan–Meier plots for progression-free survival in the intent-to-treat population

Supplementary Figure S4 Kaplan–Meier plots for overall survival in the intent-to-treat population

## Data Availability

Data underlying the findings described in this article may be obtained in accordance with AstraZeneca’s Data Sharing Policy described at https://www.astrazenecaclinicaltrials.com/our-transparency-commitments/. Data for studies directly listed on Vivli can be requested through Vivli at https://vivli.org/. Data for studies not listed on Vivli could be requested through Vivli at https://vivli.org/members/enquiries-about-studies-not-listed-on-the-vivli-platform/. An AstraZeneca Vivli member page is also available outlining further details: https://vivli.org/ourmember/astrazeneca/.
