# Supplementary material for: Durvalumab plus Paclitaxel, with or without Capivasertib or Oleclumab, in Patients with Locally Advanced/Metastatic Triple-Negative Breast Cancer
Source: Clin Cancer Res. 2026 May 26;32(16):3506–16. doi: 10.1158/1078-0432.CCR-25-4417 (PMC13473862; doi:10.1158/1078-0432.CCR-25-4417)
Supplement: Supplementary file 1 [file ccr-25-4417_supplementary_figure_s1_suppfs1.pdf]

Supplementary Fig S1. Study design.

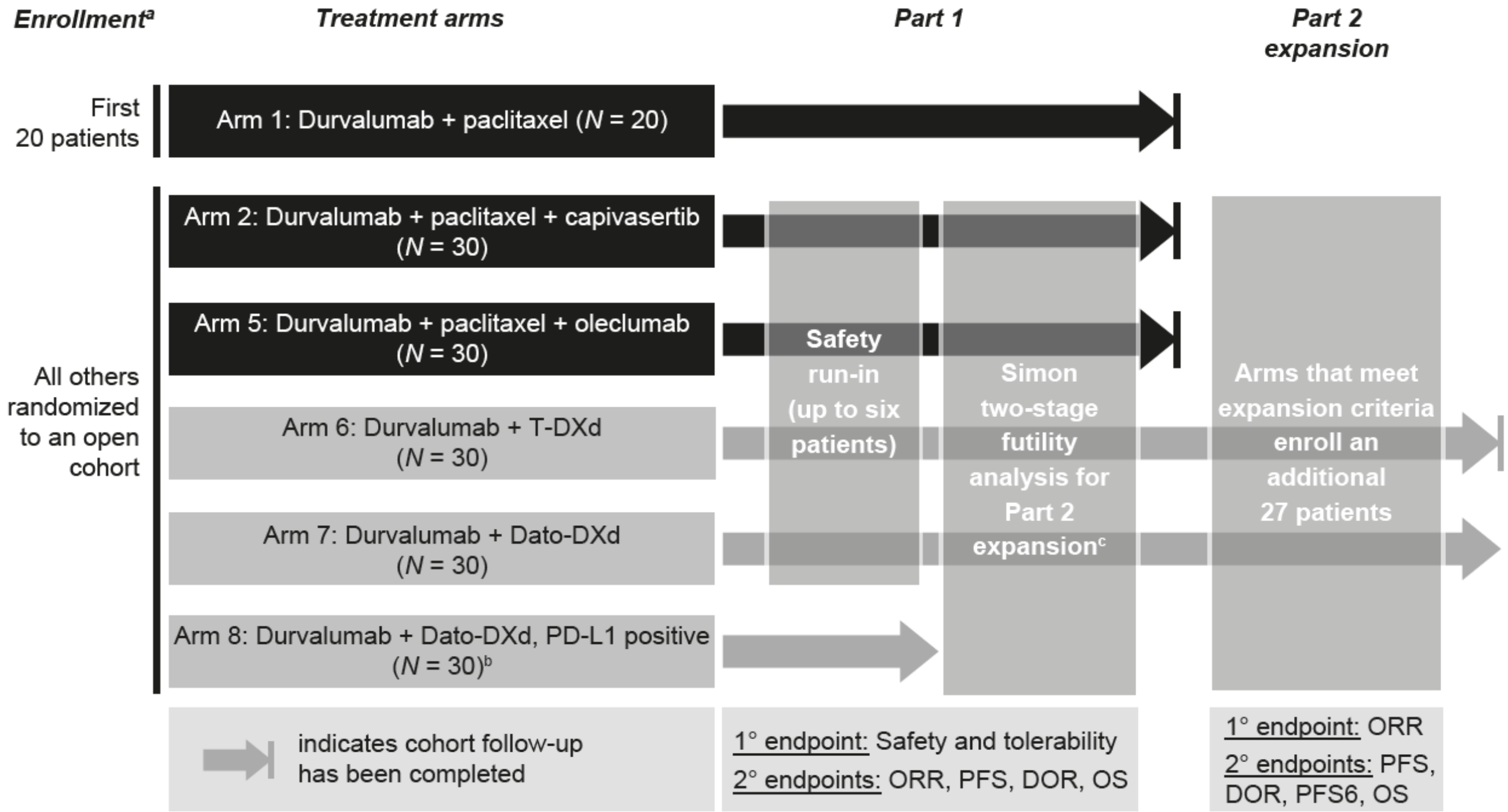

[a]Cohorts were initiated at different times between December 27, 2018, and June 15, 2023.
[b]A safety run-in will not occur for this arm as Dato-DXd + durvalumab was already evaluated and found to be tolerable, with no dose-limiting toxicities reported in arm 7.
[c]Novel treatment combinations may enter Part 2 expansion if confirmed ORR is at least 57%. If there were at least 38 of 57 evaluable patients achieving response in a treatment arm, then the data for that cohort were considered as having an adequate efficacy signal.
Dato-DXd, datopotamab deruxtecan; DOR, duration of response; ORR, objective response rate; OS, overall survival; PD-L1, programmed cell death ligand-1; PFS, progression-free survival; PFS6, progression-free survival at 6 months; T-DXd, trastuzumab deruxtecan.
