# Supplementary material for: Durvalumab plus Paclitaxel, with or without Capivasertib or Oleclumab, in Patients with Locally Advanced/Metastatic Triple-Negative Breast Cancer
Source: Clin Cancer Res. 2026 May 26;32(16):3506–16. doi: 10.1158/1078-0432.CCR-25-4417 (PMC13473862; doi:10.1158/1078-0432.CCR-25-4417)
Supplement: Supplementary file 2 [file ccr-25-4417_supplementary_figure_s2_suppfs2.pdf]

**Supplementary Fig S2.** Patient disposition in three arms from BEGONIA.

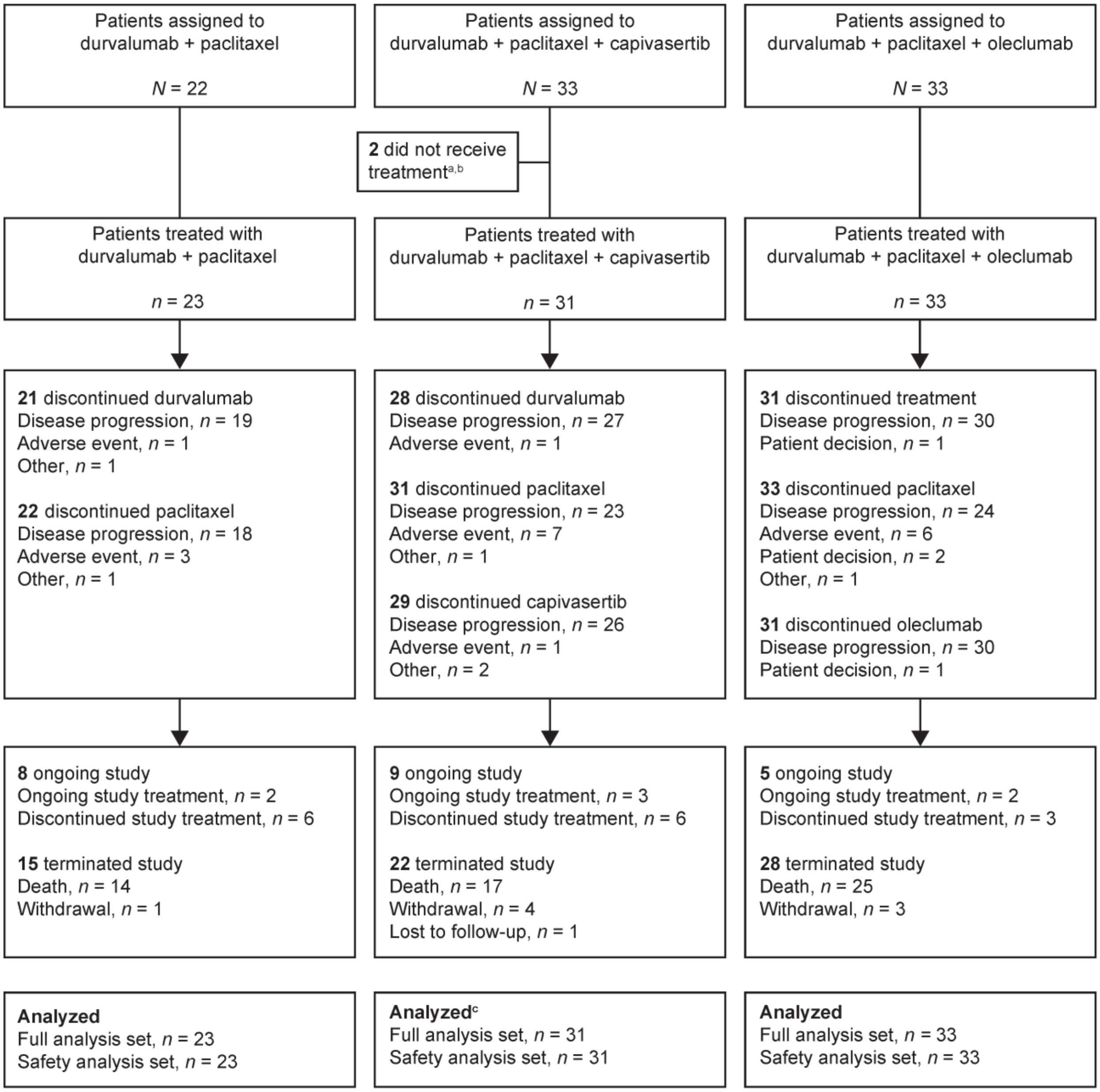


All values reported are the number of patients. Patients may appear more than once if they discontinued study treatment for more than one reason.

[a]One patient received durvalumab plus paclitaxel only after being assigned to the durvalumab plus capivasertib and paclitaxel arm and was included in the safety analysis set of the durvalumab and paclitaxel arm.

[b]One patient in the durvalumab plus capivasertib and paclitaxel arm did not receive treatment and was excluded from the safety analysis set.

[c]One patient from the durvalumab plus capivasertib and paclitaxel arm received additional treatment with oleclumab in error. This patient is included in the durvalumab plus capivasertib and paclitaxel full analysis set and safety analysis set.
