# Supplementary material for: Durvalumab plus Paclitaxel, with or without Capivasertib or Oleclumab, in Patients with Locally Advanced/Metastatic Triple-Negative Breast Cancer
Source: Clin Cancer Res. 2026 May 26;32(16):3506–16. doi: 10.1158/1078-0432.CCR-25-4417 (PMC13473862; doi:10.1158/1078-0432.CCR-25-4417)
Supplement: Supplementary file 3 [file ccr-25-4417_supplementary_figure_s3_suppfs3.pdf]

Supplementary Fig S3. Kaplan–Meier plots for progression-free survival in the intent-to-treat population.

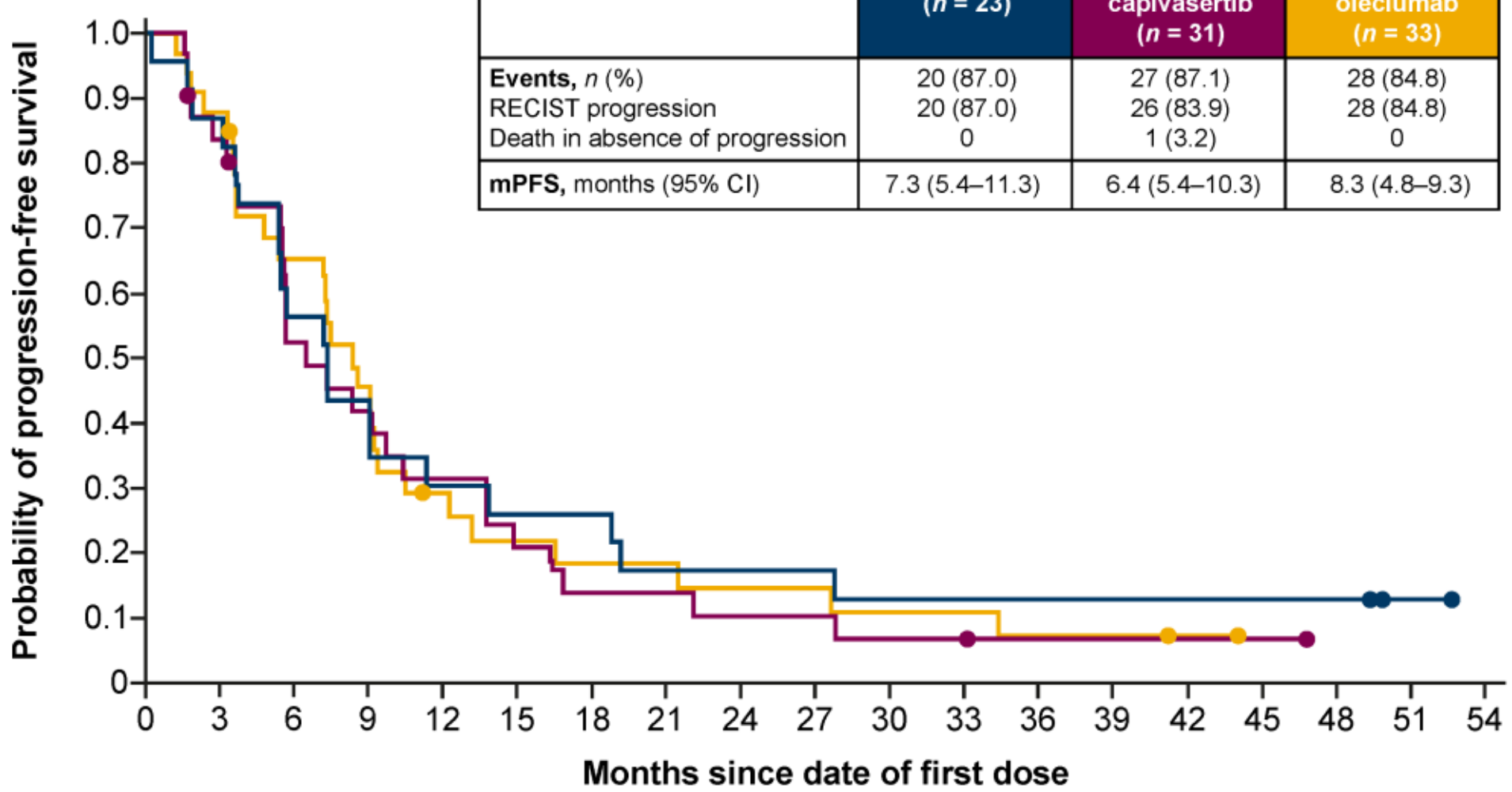

	Durvalumab + paclitaxel (*n* = 23)	Durvalumab + paclitaxel + capivasertib (*n* = 31)	Durvalumab + paclitaxel + oleclumab (*n* = 33)
**Events,** *n* (%)	20 (87.0)	27 (87.1)	28 (84.8)
RECIST progression	20 (87.0)	26 (83.9)	28 (84.8)
Death in absence of progression	0	1 (3.2)	0
**mPFS,** months (95% CI)	7.3 (5.4–11.3)	6.4 (5.4–10.3)	8.3 (4.8–9.3)

**No. at risk**	0	3	6	9	12	15	18	21	24	27	30	33	36	39	42	45	48	51	54
Durvalumab + paclitaxel	23	20	13	10	7	6	6	4	4	4	3	3	3	3	3	3	3	1	0
Durvalumab + paclitaxel + capivasertib	31	25	15	12	9	6	4	4	3	3	2	2	1	1	1	1	0	0	0
Durvalumab + paclitaxel + oleclumab	33	29	20	14	8	6	5	5	4	4	3	3	2	2	1	0	0	0	0

mPFS was calculated using the Kaplan–Meier method. Circles indicate censored observations.

CI, confidence interval; mPFS, median progression-free survival; RECIST, Response Evaluation Criteria in Solid Tumors.
