# Supplementary material for: Durvalumab plus Paclitaxel, with or without Capivasertib or Oleclumab, in Patients with Locally Advanced/Metastatic Triple-Negative Breast Cancer
Source: Clin Cancer Res. 2026 May 26;32(16):3506–16. doi: 10.1158/1078-0432.CCR-25-4417 (PMC13473862; doi:10.1158/1078-0432.CCR-25-4417)
Supplement: Supplementary file 4 [file ccr-25-4417_supplementary_figure_s4_suppfs4.pdf]

Supplementary Fig S4. Kaplan–Meier plots for overall survival in the intent-to-treat population.

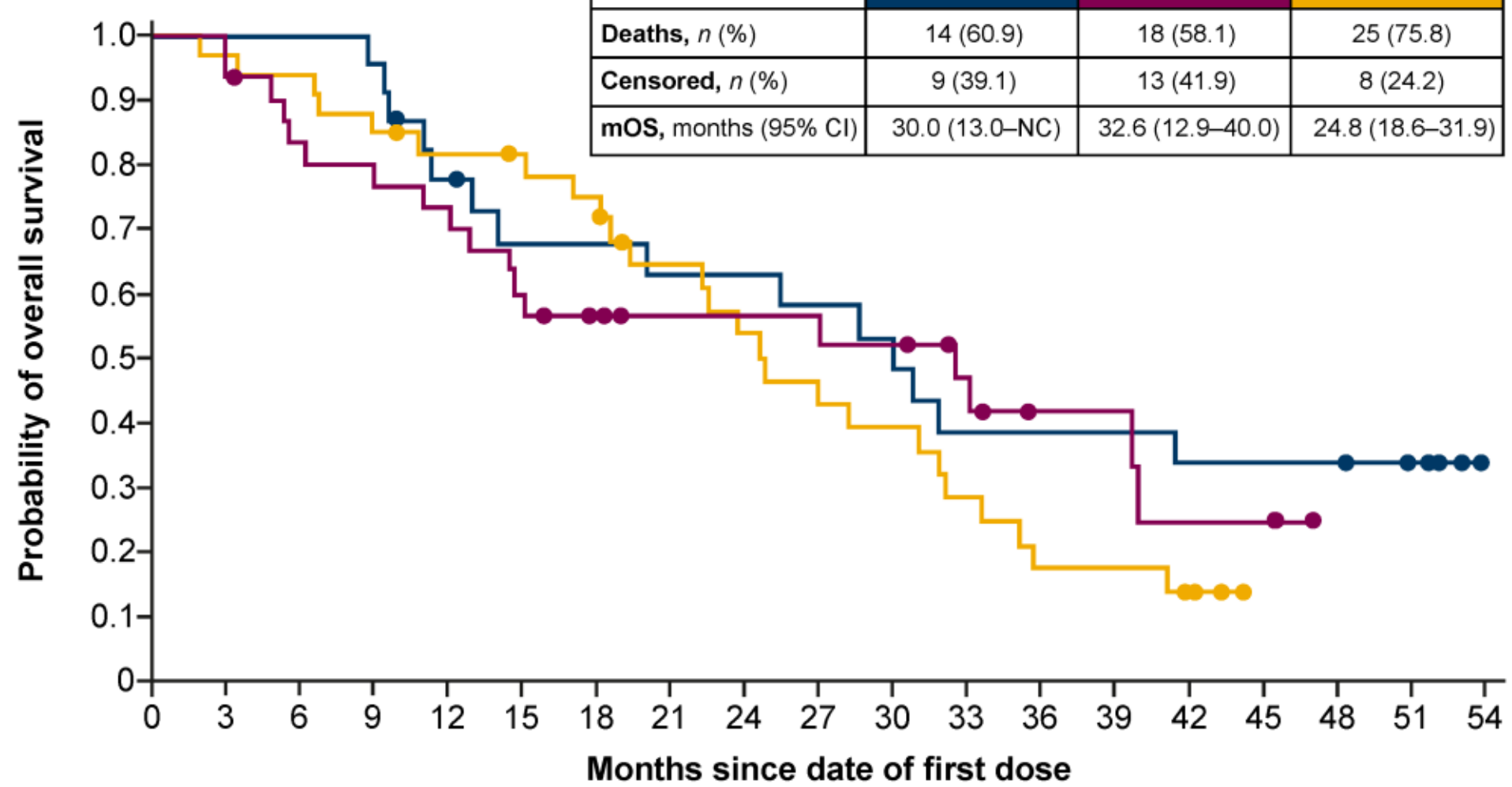

	Durvalumab + paclitaxel (*n* = 23)	Durvalumab + paclitaxel + capivasertib (*n* = 31)	Durvalumab + paclitaxel + oleclumab (*n* = 33)
**Deaths,** *n* (%)	14 (60.9)	18 (58.1)	25 (75.8)
**Censored,** *n* (%)	9 (39.1)	13 (41.9)	8 (24.2)
**mOS,** months (95% CI)	30.0 (13.0–NC)	32.6 (12.9–40.0)	24.8 (18.6–31.9)

No. at risk	0	3	6	9	12	15	18	21	24	27	30	33	36	39	42	45	48	51	54
Durvalumab + paclitaxel	23	23	23	22	17	14	14	13	13	12	11	8	8	8	7	7	7	4	0
Durvalumab + paclitaxel + capivasertib	31	31	25	24	22	18	15	13	13	13	12	9	5	5	3	3	0	0	0
Durvalumab + paclitaxel + oleclumab	33	32	31	28	26	25	23	18	15	12	11	8	5	5	3	0	0	0	0

mOS was calculated using the Kaplan–Meier method. Circles indicate censored observations.

CI, confidence interval; mOS, median overall survival; NC, not calculable.
