# Supplementary material for: Durvalumab plus Paclitaxel, with or without Capivasertib or Oleclumab, in Patients with Locally Advanced/Metastatic Triple-Negative Breast Cancer
Source: Clin Cancer Res. 2026 May 26;32(16):3506–16. doi: 10.1158/1078-0432.CCR-25-4417 (PMC13473862; doi:10.1158/1078-0432.CCR-25-4417)
Supplement: Supplementary file 5 [file ccr-25-4417_supplementary_table_s1_suppts1.pdf]

Supplementary Table S1. Representativeness of study participants.

Characteristics	Consideration
Sex	Breast cancer is the most common malignancy diagnosed in women worldwide, with more than 2.3 million new cases reported annually (1). TNBC, which accounts for 15% to 20% of all breast cancers, rarely affects men (2).
Age	The average age at the time of TNBC diagnosis is 54 years (3).
Race/ethnicity	In the USA from 2010 to 2019, the overall age-adjusted incidence rate for TNBC was 33.8 cases per 100,000 among Black women and 17.5 cases per 100,000 among White women (4).
Geography	TNBC is common among populations of African ancestry, with rates in West Africa around 46% (5). In Asia, high rates of TNBC were observed in the Indian population (6). In Europe, high proportions of TNBC cases were seen in Poland (7).
Biomarker (PD-L1)	In patients with TNBC, PD-L1 expression varies across settings, with clinical trials reporting approximately 38% of the patients PD-L1-positve (CPS ≥10) (9), whereas real-world data suggest a lower prevalence of around 27% (10).
Other considerations	Despite the high incidence of TNBC among Black women, their participation in trials remains low (8).
Overall representativeness of this study	The BEGONIA study was conducted at multiple centers in the United States, Canada, Poland, the United Kingdom, the Republic of Korea, and Taiwan. All eligible participants enrolled in the trial were female. The median age (51-54 years) reported in our study was similar to the average age distribution of TNBC in the literature. Overall, the study cohorts were generally representative of the broader TNBC population; however, Black or African American participants were underrepresented. In contrast, Asian participants were overrepresented in the durvalumab plus paclitaxel arm. Additionally,

there was a lower prevalence of patients with PD-L1–positive tumors in BEGONIA.

---

1. Bray F, Laversanne M, Sung H, Ferlay J, Siegael RL, Soerjomataram I, et al. Global cancer statistics 2022: GLOBOCAN estimates of incidence and mortality worldwide for 36 cancers in 185 countries. CA Cancer J Clin 2024;74:229-63.
2. Gucalp A, Traina TA, Eisner JR, Parker JS, Selitsky SR, Park BH, et al. Male breast cancer: a disease distinct from female breast cancer. Breast Cancer Res Treat 2019;173:37-48.
3. National Breast Cancer Foundation. Triple negative breast cancer. [cited 2025 October 30.] Available from: https://www.nationalbreastcancer.org/triple-negative-breast-cancer/#:~:text=The%20average%20age%20of%20a%20TNBC%20diagnosis%20is,breast%20cancer%20than%20other%20types%20of%20breast%20cancer.
4. Du XL, Li Z. Incidence trends in triple-negative breast cancer among women in the United States from 2010 to 2019 by race/ethnicity, age and tumor stage. Am J Cancer Res 2023;13:678-91.
5. Hercules SM, Alnajar M, Chen C, Mladjenovic SM, Shipeolu BA, Perkovic O, et al. Triple-negative breast cancer prevalence in Africa: a systematic review and meta-analysis. BMJ Open 2022;12:e055735.
6. Kulkarni A, Kelkar DA, Parikh N, Shashidhara LS, Koppiker CB, Kulkarni M. Meta-analysis of prevalence of triple-negative breast cancer and its clinical features at incidence in Indian patients with breast cancer. JCO Glob Oncol 2020;6:1052-62.
7. Rosińska M, Dubiański R, Konieczna A, Poleszczuk J, Pawlik H, Nowecki ZI, et al. Retrospective observational study to determine the epidemiology and treatment patterns of patients with triple-negative breast cancer. Cancers (Basel) 2024;16:1087.
8. Rao D, Brabant P, Chau I, Darrell M, Yao C-Y, Roth M, et al. Racial disparities among triple negative breast cancer clinical trial enrollees between 2010-2023. J Clin Oncol 2024;42:1093.
9. Cortes J, Cescon DW, Rugo HS, Nowecki Z, Im SA, Yusof MM, et al. Pembrolizumab plus chemotherapy versus placebo plus chemotherapy for previously untreated locally recurrent inoperable or metastatic triple-negative breast cancer (KEYNOTE-355): a randomised, placebo-controlled, double-blind, phase 3 clinical trial. Lancet 2020;396:1817-28.
10. Sigurjonsdottir G, De Marchi T, Ehinger A, Hartman J, Bosch A, Staaf J, Killander F, Niméus E. Comparison of SP142 and 22C3 PD-L1 assays in a population-based cohort of triple-negative breast cancer patients in the context of their clinically established scoring algorithms. Breast Cancer Res. 2023;25(1):123.
