# Supplementary material for: Durvalumab plus Paclitaxel, with or without Capivasertib or Oleclumab, in Patients with Locally Advanced/Metastatic Triple-Negative Breast Cancer
Source: Clin Cancer Res. 2026 May 26;32(16):3506–16. doi: 10.1158/1078-0432.CCR-25-4417 (PMC13473862; doi:10.1158/1078-0432.CCR-25-4417)
Supplement: Supplementary file 6 [file ccr-25-4417_supplementary_table_s2_suppts2.pdf]

Supplementary Table S2. Summary of exposure, treatment delays, and dose reductions.

Exposure parameter	Durvalumab + paclitaxel (*N* = 23)	Durvalumab + paclitaxel + capivasertib (*N* = 31)	Durvalumab + paclitaxel + oleclumab (*N* = 33)
Durvalumab exposure			
Median duration of treatment (range), months	9.2 (1–54)	7.4 (1–47)	8.3 (2–44)
Median number of doses received (range)	10.0 (1–59)	8.0 (1–46)	9.0 (2–49)
Patients with dose delay, *n* (%)	9 (39.1)	14 (45.2)	15 (45.5)
Patients with infusion interruptions, *n* (%)	0	0	0
Paclitaxel exposure			
Median duration of treatment (range), months	7.6 (0–54)	7.2 (1–29)	7.4 (1–23)
Median number of doses received (range)	24.0 (1–174)	21.0 (3–88)	24.0 (5–54)
Patients with dose delay, *n* (%)	9 (39.1)	15 (48.4)	10 (30.3)
Patients with dose reduction, *n* (%)	14 (60.9)	15 (48.4)	14 (42.4)
Patients with infusion interruptions, *n* (%)	0	2 (6.5)	2 (6.1)
Capivasertib exposure			
Median duration of treatment (range), months	–	6.3 (0–47)	–
Median number of doses received (range)	–	18.5 (1–141)	–
Patients with dose delay, *n* (%)	–	0	–
Patients with dose reduction, *n* (%)	–	14 (45.2)	–
Patients with dose interruption, *n* (%)	–	18 (58.1)	–
Oleclumab exposure			
Median duration of treatment (range), months	–	–	8.3 (1–44)
Median number of doses received (range)	–	–	11.0 (3–51)
Patients with dose delay, *n* (%)	–	–	11 (33.3)
Patients with dose reduction, *n* (%)	–	–	0
Patients with infusion interruptions, *n* (%)	–	–	2 (6.1)
Patients with dose interruption, *n* (%)	–	–	4 (12.1)
