# Supplementary material for: Durvalumab plus Paclitaxel, with or without Capivasertib or Oleclumab, in Patients with Locally Advanced/Metastatic Triple-Negative Breast Cancer
Source: Clin Cancer Res. 2026 May 26;32(16):3506–16. doi: 10.1158/1078-0432.CCR-25-4417 (PMC13473862; doi:10.1158/1078-0432.CCR-25-4417)
Supplement: Supplementary file 7 [file ccr-25-4417_supplementary_table_s3_suppts3.pdf]

Supplementary Table S3. Most common AEs (in ≥5% of patients in any arm) possibly related to durvalumab.

**AE category, *n* (%)**	**Durvalumab + paclitaxel (*N* = 23)**	**Durvalumab + paclitaxel + capivasertib (*N* = 31)**	**Durvalumab + paclitaxel + oleclumab (*N* = 33)**
Any AE related to durvalumab	15 (65.2)	22 (71.0)	23 (69.7)
Rash	6 (26.1)	8 (25.8)	4 (12.1)
Hypothyroidism	6 (26.1)	1 (3.2)	7 (21.2)
Fatigue	4 (17.4)	6 (19.4)	5 (15.2)
Pruritus	3 (13.0)	7 (22.6)	2 (6.1)
ALT increased	2 (8.7)	0	2 (6.1)
Lipase increased	2 (8.7)	0	3 (9.1)
Pyrexia	1 (4.3)	4 (12.9)	0
Nausea	1 (4.3)	0	3 (9.1)
Hyperthyroidism	1 (4.3)	0	2 (6.1)
Stomatitis	1 (4.3)	2 (6.5)	0
Diarrhea	0	2 (6.5)	2 (6.1)
Neutropenia	0	2 (6.5)	0
Hypertransaminasemia	0	2 (6.5)	0
Rash maculopapular	0	2 (6.5)	0
Myalgia	0	2 (6.5)	1 (3.0)
Dermatitis acneiform	0	0	2 (6.1)
Amylase increased	0	0	2 (6.1)

Abbreviations: AE, adverse event; ALT, alanine aminotransferase.
