# Supplementary material for: Durvalumab plus Paclitaxel, with or without Capivasertib or Oleclumab, in Patients with Locally Advanced/Metastatic Triple-Negative Breast Cancer
Source: Clin Cancer Res. 2026 May 26;32(16):3506–16. doi: 10.1158/1078-0432.CCR-25-4417 (PMC13473862; doi:10.1158/1078-0432.CCR-25-4417)
Supplement: Supplementary file 8 [file ccr-25-4417_supplementary_table_s4_suppts4.pdf]

Supplementary Table S4. Most common AEs (in ≥5% of patients) possibly related to capivasertib.

**AE category, *n* (%)**	**Durvalumab + paclitaxel + capivasertib (*N* = 31)**
Any AE related to capivasertib	27 (87.1)
Diarrhea	20 (64.5)
Rash	15 (48.4)
Hyperglycemia	9 (29.0)
Pruritus	8 (25.8)
Nausea	5 (16.1)
Rash maculopapular	4 (12.9)
Fatigue	4 (12.9)
Pyrexia	4 (12.9)
Stomatitis	3 (9.7)
Abdominal pain	2 (6.5)
Flatulence	2 (6.5)
Vomiting	2 (6.5)
AST increased	2 (6.5)

Abbreviations: AE, adverse event; AST, aspartate aminotransferase.
