# Supplementary material for: Durvalumab plus Paclitaxel, with or without Capivasertib or Oleclumab, in Patients with Locally Advanced/Metastatic Triple-Negative Breast Cancer
Source: Clin Cancer Res. 2026 May 26;32(16):3506–16. doi: 10.1158/1078-0432.CCR-25-4417 (PMC13473862; doi:10.1158/1078-0432.CCR-25-4417)
Supplement: Supplementary file 9 [file ccr-25-4417_supplementary_table_s5_suppts5.pdf]

Supplementary Table S5. Most common AEs (in ≥5% of patients) possibly related to oleclumab.

**AE category, *n* (%)**	**Durvalumab + paclitaxel + oleclumab (*N* = 33)**
Any AE related to oleclumab	18 (54.5)
Fatigue	5 (15.2)
Nausea	4 (12.1)
Diarrhea	3 (9.1)
Amylase increased	3 (9.1)
Lipase increased	3 (9.1)
Pruritus	3 (9.1)
Hypothyroidism	2 (6.1)
Dermatitis acneiform	2 (6.1)
Rash	2 (6.1)

Abbreviation: AE, adverse event.
