# Supplementary material for: Durvalumab plus Paclitaxel, with or without Capivasertib or Oleclumab, in Patients with Locally Advanced/Metastatic Triple-Negative Breast Cancer
Source: Clin Cancer Res. 2026 May 26;32(16):3506–16. doi: 10.1158/1078-0432.CCR-25-4417 (PMC13473862; doi:10.1158/1078-0432.CCR-25-4417)
Supplement: Supplementary file 10 [file ccr-25-4417_supplementary_table_s6_suppts6.pdf]

Supplementary Table S6. Response and survival outcome per detection of *PIK3CA/AKT1/PTEN* alterations.

**Outcome**[a]	**Durvalumab + paclitaxel**	**Durvalumab + paclitaxel + capivasertib**
**With *PIK3CA/AKT1/PTEN* altered tumors**	***N* = 3**	***N* = 8**
Confirmed ORR, *n* (%)	1 (33.3)	6 (75.0)
Median PFS (95% CI), months	5.4 (3.6–NC)	10.1 (2.7–22.1)
Median DOR (95% CI), months	17.0 (NC–NC)	12.6 (3.9–NC)
Median OS (95% CI), months	31.8 (13.0–NC)	26.3 (4.8–NC)
**Without *PIK3CA/AKT1/PTEN* altered tumors**	***N* = 11**	***N* = 20**
Confirmed ORR, *n* (%)	7 (63.6)	11 (55.0)
Median PFS (95% CI), months	13.8 (3.6–NC)	8.3 (3.7–10.3)
Median DOR (95% CI), months	26.1 (5.6–NC)	7.4 (3.7–14.6)
Median OS (95% CI), months	NC (25.5–NC)	32.6 (14.5–NC)

Median DOR, PFS, and OS were calculated using the Kaplan–Meier method.
Abbreviations: CI, confidence interval; DOR, duration of response; NC, not calculable; ORR, objective response rate; OS, overall survival; PFS, progression-free survival.
[a]Nine patients in the durvalumab plus paclitaxel arm and three patients in the capivasertib combination arm had a missing *PIK3CA/AKT1/PTEN* alteration result as assessed via aggregated ctDNA and tumor tissue.
