# Supplementary material for: Durvalumab plus Paclitaxel, with or without Capivasertib or Oleclumab, in Patients with Locally Advanced/Metastatic Triple-Negative Breast Cancer
Source: Clin Cancer Res. 2026 May 26;32(16):3506–16. doi: 10.1158/1078-0432.CCR-25-4417 (PMC13473862; doi:10.1158/1078-0432.CCR-25-4417)
Supplement: Supplementary file 11 [file ccr-25-4417_supplementary_table_s7_suppts7.pdf]

Supplementary Table S7. Response and survival outcome per CD73 expression status.

**Outcome**[a]	**Durvalumab + paclitaxel**	**Durvalumab + paclitaxel + oleclumab**
CD73 positive	*N* = 10	*N* = 19
Confirmed ORR, *n* (%)	7 (70.0)	10 (52.6)
Median PFS (95% CI), months	12.6 (3.6–27.8)	8.5 (5.3–13.1)
Median DOR (95% CI), months	15.7 (3.8–NC)	8.5 (5.0–25.8)
Median OS (95% CI), months	NC (11.0–NC)	23.7 (10.7–35.1)
CD73 negative	*N* = 7	*N* = 5
Confirmed ORR, *n* (%)	2 (28.6)	1 (20.0)
Median PFS (95% CI), months	5.4 (1.9–7.3)	4.8 (3.6–NC)
Median DOR (95% CI), months	10.4 (3.7–NC)	3.7 (NC–NC)
Median OS (95% CI), months	28.6 (9.6–NC)	24.8 (17.1–NC)

Median PFS and OS were calculated using the Kaplan–Meier method.

Abbreviations: CI, confidence interval; NC, not calculable; ORR, objective response rate; OS, overall survival; PFS, progression-free survival.

[a]Six patients in the durvalumab and paclitaxel arm and nine patients in the oleclumab combination arm had missing CD73 expression status, as assessed via immunohistochemistry. Expression was defined by the percentage of stromal immune cells with CD73 detected. A sample was considered positive if expression was ≥1%, and negative if it was <1%.
